# Effect of Several Nutrients and Environmental Conditions on Intracellular Melatonin Synthesis in *Saccharomyces cerevisiae*

**DOI:** 10.3390/microorganisms8060853

**Published:** 2020-06-05

**Authors:** María Ángeles Morcillo-Parra, Gemma Beltran, Albert Mas, María-Jesús Torija

**Affiliations:** Grup de Biotecnologia Enològica, Departament de Bioquímica i Biotecnologia, Facultat d’Enologia, Universitat Rovira i Virgili, c/Marcel·lí Domingo, 1-43007 Tarragona, Spain; maangeles.morcillo@urv.cat (M.Á.M.-P.); gemma.beltran@urv.cat (G.B.); albert.mas@urv.cat (A.M.)

**Keywords:** melatonin, alcoholic fermentation, yeast, temperature, *Saccharomyces cerevisiae*

## Abstract

Melatonin is a bioactive compound that is present in fermented beverages and has been described to be synthesized by yeast during alcoholic fermentation. The aim of this study was to assess the capacity of intracellular and extracellular melatonin production by different *Saccharomyces* strains from diverse food origin and to study the effects of different fermentation parameters, such as sugar and nitrogen concentration, temperature or initial population, on melatonin production using a synthetic grape must medium. Melatonin from fermentation samples was analyzed by liquid chromatography mass spectrometry. Intracellular melatonin synthesis profile did not present differences between yeast strains. However, extracellular melatonin production depended on the yeast origin. Thus, we suggest that melatonin production and secretion during the different yeast growth phases follows a species-specific pattern. Other parameters that affected the fermentation process such as sugar content and low temperature had an impact on intracellular melatonin production profile, as well as the melatonin content within the cell. This study reports the effect of several conditions on the melatonin synthesis profile, highlighting its possible role as a signal molecule.

## 1. Introduction

Several factors, either biotic or abiotic, can affect the yeasts present during alcoholic fermentation, and thus, modify the fermentation performance and the production of bioactive compounds [1,2]. The microbial interactions and responses, such as the synthesis of antimicrobial compounds, competition for nutrients and yeast–yeast cell contact are the main biotic factors [3,4]. On the other hand, abiotic factors are defined as environmental aspects that affect the winemaking process, such as nutrients (sugar and nitrogen), temperature and pH, among others [5]. Sugar content in grape must is the one of the first stresses that yeasts have to deal with. This high sugar content derives in osmotic shock, and the yeast response is to modify the cell wall and the cytoskeleton as well as to activate the synthesis of glycerol to reestablish the osmotic balance [6,7]. Nitrogen is also an important and critical nutrient for yeast cells. In fact, low nitrogen levels can result in stuck or sluggish fermentation [8]. Furthermore, temperature can also alter the development of fermentation, specifically yeast growth [9,10].

Melatonin is a bioactive molecule that has been recently described to have a positive role against oxidative stress [11,12] and UV stress [13] in yeasts. In addition to its protective role, melatonin is produced by yeast during the winemaking process [14,15,16,17].

Since Sprenger and collaborators [18] observed intracellular melatonin after tryptophan pulse when yeast cells were arrested in minimal medium, many studies have focused on finding this molecule in fermented beverages such as wine or beer [14,19,20,21]. Rodriguez-Naranjo and collaborators [22] highlighted the role of yeast, specifically *Saccharomyces cerevisiae*, in the production of melatonin during the winemaking process. However, not only does *S. cerevisiae* produce melatonin but also several non-*Saccharomyces* strains are able to synthetize melatonin during alcoholic fermentation [14,15,16,23,24,25]. Melatonin production depends on the availability of tryptophan, which is a precursor [14,16,26], but also some tryptophan derivatives (N-acetyl serotonin or 5-methoxytryptamine) can be used as melatonin precursors [27]. However, other fermentation conditions, such as growth medium composition (reducing sugars or nitrogen content) and yeast growth phase, can affect melatonin synthesis [25,26]. Moreover, melatonin content is positively correlated with the ethanol production rate during alcoholic fermentation, suggesting that melatonin could participate in alcoholic fermentation [28].

Melatonin has been described in the extracellular medium during the exponential phase of growth (between the first and second day) [14,15,17,23,24,26] and at the end of alcoholic fermentation (at the stationary phase) [25] depending on the yeast species involved in the fermentation. Nevertheless, few studies about intracellular melatonin have been published. Some authors have provided evidence that melatonin is present intracellularly after precursor pulses [16,18,27], and also, in oenological conditions, both in *Saccharomyces* [17] and in non-*Saccharomyces* yeasts [16,24], although few strains have been studied.

The aim of this study was (i) to assess the capacity of intra- and extracellular melatonin production by different strains of *Saccharomyces* yeasts and (ii) to study the effects of different nutrient and environmental conditions (sugar, nitrogen, temperature, inoculum and cell synchronization) on melatonin production in order to understand the conditions required for the production of melatonin by yeast.

## 2. Materials and Methods

### 2.1. Yeast Strain and Inoculum Preparation

In this study, we used three different strains of *S. cerevisiae*, QA23 (from wine), Instaferm (from bread baking) and Levucell SC20 (from animal nutrition), and one strain of *S. pastorianus*, Diamond (from beer), which were provided by Lallemand S.A. (Lallemand Inc. Montreal, Canada). The synchronization experiments were performed on the derivative haploid of the commercial strains QA23 (QA23 ho-, [29]). Dry yeasts were rehydrated in water at 37 °C for 30 min. Afterwards, the precultures were prepared in 50 mL of YPD broth (1% (*w*/*v*) yeast extract, 2% (*w*/*v*) glucose and 2% (*w*/*v*) bacteriological peptone (Panreac Quimica SLU, Barcelona, Spain)) at 28 °C with a stirring rate of 120 rpm in an orbital shaker. Then, yeast cells were transferred into fresh minimal medium (YNB; 1X yeast nitrogen base without amino acids or ammonia (Becton, Dickinson and Company, Sparks, MD, USA), 2% (*w*/*v*) glucose, and 350 mM (NH_4_)_2_ SO_4_ (Panreac Quimica SLU, Barcelona, Spain)) and cultured for three days at 28 °C and 120 rpm.

### 2.2. Alcoholic Fermentation Conditions

Synthetic grape must was prepared as in Beltran et al. [30], but with a modification on the aromatic amino acid (tryptophan, tyrosine and phenylalanine) concentration. In this study, a five-fold increase in aromatic amino acids was used at the expense of the remaining amino acids to maintain the concentration of yeast assimilable nitrogen (YAN) at 300 mg N/L. This synthetic grape must (with 200 g/L sugars (1:1 glucose:fructose) and 300 mg N/L) was considered the standard medium for this study. After the preparation, the synthetic grape must was stored at 4 °C until used. In higher temperature experiments (12 and 28 °C), the must was pre-warmed to the desired temperature before inoculation.

Two different experiments were designed. The first consisted of performing fermentations in standard medium using different *S. cerevisiae* strains. Yeasts (QA23, Instaferm, Levucell and Diamond) were inoculated at 2 × 10^6^ cell/mL in 450 mL of synthetic must and incubated in 500-mL bottles with orbital agitation (120 rpm) at 28 °C. Fermentations were carried out in triplicate. Sampling was done at different time points of the growth phases. For harvesting, 10 OD cultures (equivalent to 10^8^ cells) were centrifuged at 7800 rpm for 5 min in 50-mL centrifuge tubes (Sigma 2-16, Rotor 12151, Germany). Pellets were washed with Milli-Q water, frozen with liquid nitrogen and stored at −80 °C until melatonin analysis. Supernatants were stored at −20 °C until melatonin analysis was performed.

In the second experiment, different fermentation parameters were modified, using the conditions implemented with the QA23 strain in the first experiment (200 g/L, 300 mg N/L) and 10^6^ cell/mL and 28 °C as control fermentations. From precultures in YPD and YNB, the QA23 strain was inoculated in 45 mL of standard medium in 50-mL flasks. All the conditions were tested by triplicate. To study the effect of nutrients (sugar and nitrogen) on melatonin production, the final concentration of sugars and assimilable nitrogen were decreased in some fermenters to 20 g/L and 100 mg N/L, respectively. To analyze the effect of environmental parameters, inoculum size was fixed at 10^7^ and 10^8^ cell/mL, and fermentation temperature was adjusted to 4 or 12 °C. Cell cycle synchronization (arrested and not-arrested cells) was performed by adding alpha factor (5 µg/mL (Sigma Aldrich, St. Louis, MO, USA)), in the haploid QA23, before inoculation in grape must.

In all fermentations, samples (10 OD) for intracellular melatonin determination were taken every 10 min (except for synchronization, for which time points were every 20 min) and centrifuged at 7800 rpm for 3 min in 50-mL centrifuge tubes (Sigma 2-16, Rotor 12151, Germany). After centrifugation, pellets were washed with Milli-Q water, frozen with liquid nitrogen and stored at −80 °C until melatonin analysis was performed.

### 2.3. Melatonin Analysis

Melatonin samples were prepared and analyzed as described in Morcillo-Parra et al. [17]. Briefly, intracellular metabolites were extracted by adapting a boiling-buffered ethanol method [31], in which cells were incubated with 1 mL of a solution of 75% (*v*/*v*) boiling absolute ethanol containing 70 mM HEPES buffer (pH 7.5) for 3 min at 80 °C, then, concentrated by evaporation at 45 °C in a SpeedBack (Concentrator plus, Eppendorf Ibérica, Madrid, Spain), and resuspended in 1 mL of Milli-Q water and centrifuged for 10 min at 5000 rpm. Finally, the supernatant was transferred to a new tube and stored at −20 °C until use.

Both extracellular and intracellular melatonin samples were extracted with a chloroform method. In brief, 50 µL of sample was mixed with Milli-Q water (1:1, v:v). Then, 500 µL of chloroform was added, and the mixture was shaken for 1 h at 1200 rpm. The organic phase was dried under a flow of nitrogen gas and resuspended in 50 µL of a methanol and water mixture (40:60, v:v). Then, samples were centrifuged for 5 min at 14,500 rpm and analyzed by performing liquid chromatography mass spectrometry (LC-MS/MS) as described in Morcillo-Parra et al. [17]. The system was based on high-performance liquid chromatography coupled to a triple quadrupole mass spectrometer (Agilent G6410; Agilent Technologies, Palo Alto, CA, USA), using an Agilent 150 × 2.1 mm i.d., 3.5 µM, Zorbax Sb-Aq column in a binary gradient consisting of (A) water and (B) methanol as solvents, both containing 0.1% (*v*/*v*) formic acid. The elution profile was 100% B (4 min), 10% B (6 min). The temperature was set at 40 °C, the flow rate was 0.4 mL/min and the injection volume was 7 µL. The ESI conditions were as follows: a drying gas temperature of 30 °C, a flow of 12 L/min, and a nebulizer gas pressure of 25 psi. The capillarity voltage was set up at 2500 V. Acquisition was done in positive polarity. Triple quadrupole operated in multiple reaction monitoring mode, applying a fragmentor voltage of 135 V and a cell accelerator voltage of 65 V.

Melatonin quantification was performed using Agilent MassHunter WorkStation Software Quantitative Analysis Version B0104 (Agilent Technologies, Palo Alto, CA, USA) by comparing the 233/174 transition MS data of the sample and the standard.

### 2.4. Statistical Analysis

Data are expressed as the mean and standard deviation of triplicates. ANOVA and Fisher and Tukey’s test analysis using XLSTAT 2020 software (Addinsoft, New York, NY, USA) were performed to evaluate the effect of each condition in melatonin synthesis (Tables S5–S10). The results were considered statistically significant at a *p*-value lower than 0.05. The GraphPad Prism 7 program (GraphPad software, San Diego, CA, USA) was used for graphical data modeling.

## 3. Results and Discussion

### 3.1. Effect of Strain on Melatonin Synthesis

To determine whether melatonin production during alcoholic fermentation is a general trait of *Saccharomyces* strains or if instead it is a strain-dependent trait or linked to the strain origin, fermentations with four strains from different environments (QA23, a wine yeast; Instaferm, a baking yeast; Levucell, an animal food yeast; Diamond, a beer yeast) were carried out.

**Figure 1:**
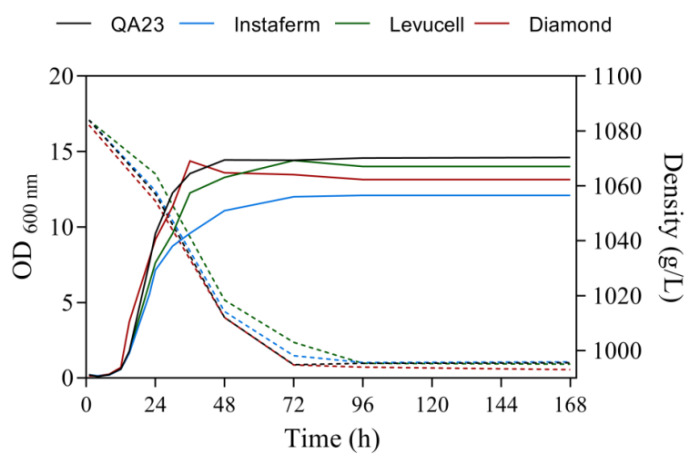
Fermentation kinetics of the different *Saccharomyces* yeast strains by monitoring the density of the must (dotted line) and yeast population, measured as optical density at 600 nm (OD_600_, solid line) throughout the fermentation.

In fact, some differences were observed in terms of growth during alcoholic fermentation (Figure 1). All yeast strains presented similar growth, except the Instaferm strain, which had lower maximal growth. However, all growth phases occurred at similar time points. In terms of fermentation performance, Diamond and QA23 consumed all sugar after three days of fermentation, as observed in the study performed by Lleixà et al. [32] under similar conditions, while Levucell and Instaferm needed one more day to consume all sugar. Both yeast strains are not used for ethanol production, which explains this slower fermentative behavior. These results were similar to those of Albertin et al. [33], who described that wine strains in enology medium are able to consume all sugars, while other food origin yeasts displayed slow or incomplete fermentations.

**Figure 2:**
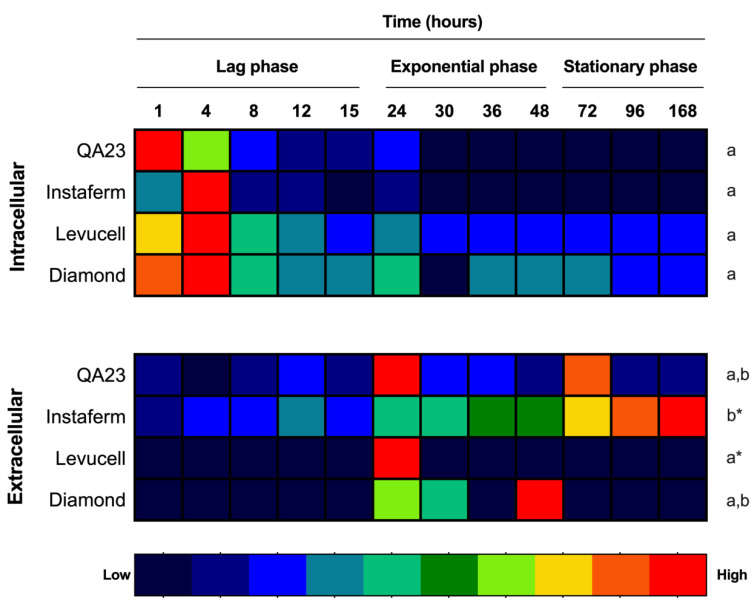
Heatmap of melatonin production by the four *Saccharomyces* strains during the different yeast growth phases. Concentrations were normalized to its maximum level. Different letters indicate significant differences between conditions within each strain, *p* < 0.05.

In addition to the fermentation kinetics, intracellular and extracellular melatonin were measured by LC-MS/MS. On one hand, melatonin was produced intracellularly during the lag phase of yeast growth (Figure 2, Table S1), with QA23 showing the fastest synthesis (1 h), whereas the other *Saccharomyces* yeasts reached their maximum at 4 h, as we had already observed in *Saccharomyces* and non-*Saccharomyces* yeasts in similar conditions [17,24].

**Figure 3:**
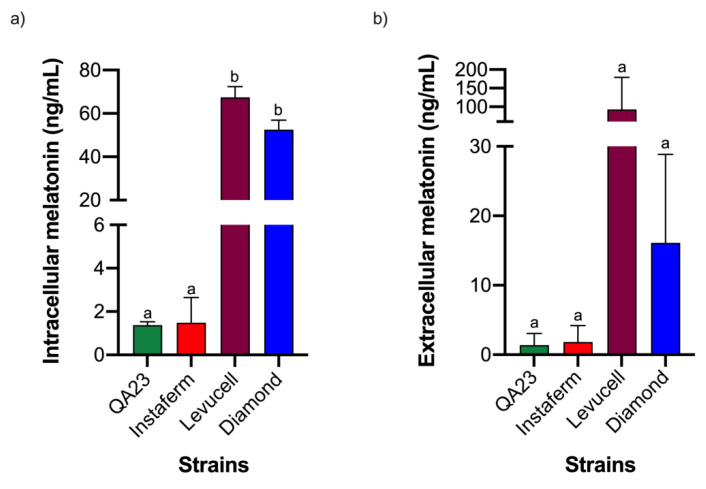
Maximal intracellular (**a**) and extracellular (**b**) melatonin concentration by different *Saccharomyces* strains. Intracellular melatonin is expressed in ng/mL per 10 OD. Error bars represent ± SD of *n* = 3 by ANOVA. Different letters indicate significant differences between strains. *p* < 0.05.

Even though melatonin peaks appeared at different time points (1 and 4 h), it is important to highlight that intracellular melatonin was observed during lag phase in all yeast strains, reinforcing the relationship of melatonin with the yeast growth curve and yeast adaptation to the medium, as previously described by Rodríguez-Naranjo et al. [26]. In fact, in that study, melatonin was proposed as a signal molecule in yeast cells, linked to yeast growth phases and medium adaptation, even though it was only based on extracellular melatonin [26]. Although all yeast strains produced intracellular melatonin with a similar profile, melatonin quantities clearly differed between strains (Figure 3, Tables S1, S5 and S6), with Levucell and Diamond synthesizing more melatonin and presenting a basal concentration throughout all fermentations (Figure 3a). Additionally, extracellular melatonin was detected during alcoholic fermentation in all *Saccharomyces* strains tested (Figure 2). The QA23 and Levucell strains reached their maximum level at 24 h, confirming previous results [17,25,26]. However, in the QA23 strain, the quantity of melatonin decreased from this point until 72 h when a synthesis rebound was observed, whereas in the Levucell strain no more melatonin was detected throughout fermentation. Conversely, Diamond exhibited its extracellular melatonin peak later, at 48 h. However, a smaller peak was also detected at 24 h, similar to QA23 and Levucell strains. Finally, in the Instaferm strain, melatonin content progressively increased until the end of fermentation (at 7 days), being the only one that accumulated melatonin during fermentation. These results were similar to those reported by Fernández-Cruz et al. [34], in which six *Saccharomyces* strains produced melatonin at different points of alcoholic fermentation.

Although the extracellular melatonin concentrations on the maximal point might reproduce the differences observed in the intracellular concentration, the huge variability observed did not allow to set statistical significance (Figure 3b, Tables S1, S7 and S8). This variability is mainly due to the fast synthesis and disappearance of this molecule, which is sometimes difficult to detect with precision. 

**Figure 4:**
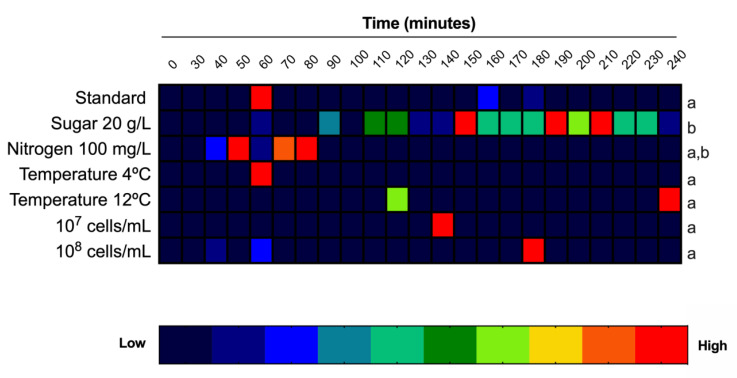
Heatmap of melatonin production in several fermentation conditions in *S. cerevisiae* QA23: Low sugar (20 g/L), low nitrogen (100 mg N/L) concentration, different temperatures and inoculum amount. Concentrations were normalized to its maximum level. Standard fermentation was grape must with 200 g/L sugar and 300 mg N/L, inoculated at 10^6^ cells/mL and fermented at 28 °C. Different letters indicate significant differences between conditions, *p* < 0.05.

As intracellular melatonin synthesis occurred during lag phase, and we proposed that this synthesis was related to the adaptation of yeast cells to the medium, we modified different important fermentation parameters, such as sugar and nitrogen concentrations, temperature or initial population, to determine if any of these parameters were responsible for triggering melatonin synthesis. Intracellular melatonin was monitored during the first 4 h of growth, covering the part of lag phase in which intracellular synthesis was detected in the previous experiment. The results were expressed as a heat map (Figure 4).

### 3.2. Effects of Sugar and Nitrogen on Melatonin Synthesis

**Figure 5:**
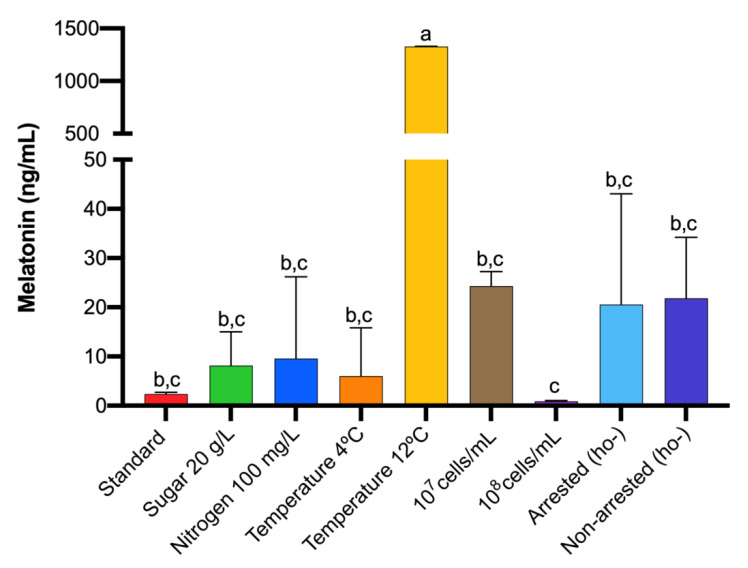
Maximal intracellular melatonin concentration by *S. cerevisiae* QA23 in several fermentation conditions: Low sugar (20 g/L), low nitrogen (100 mg N/L) concentration, temperature, inoculum amount, arrested and non-arrested cells. Data are expressed in ng/mL per 10^8^ cells. Standard fermentation was grape must with 200 g/L sugar and 300 mg N/L, inoculated at 10^6^ cells/mL and fermented at 28 °C. Error bars represent ± SD of *n* = 3 by ANOVA. Different letters indicate significant differences between conditions, *p* < 0.05.

As explained in the previous experiment, QA23 in standard conditions (200 g/L sugar and 300 mg N/L) showed a peak on intracellular melatonin at 60 min (Figure 4). However, in low-sugar conditions (20 g/L), intracellular melatonin was detected starting 90 min after inoculation and increasing progressively until 150, 190 and 210 min, when the maximum melatonin concentration was quantified. In contrast, in low-nitrogen conditions, intracellular melatonin had a similar profile as that in standard conditions, appearing in a similar timeframe (50 and 80 min), although it remained longer in the intracellular medium. Even though we observed differences in the time of detection, in which intracellular melatonin is synthetized, we did not observe those differences in the amount of melatonin produced, again due to the high dispersion between triplicates in some samples (Figure 5, Tables S2, S9 and S10). Thus, only glucose limitation affected melatonin synthesis profile during alcoholic fermentation. This result may highlight osmotic stress as being responsible for intracellular melatonin synthesis, but also a change in the carbon metabolism of yeast due to the low sugar concentration. In fact, recent results of our group have shown that melatonin interacts with some glycolytic proteins in yeast with high fermentative capacity, pinpointing a possible role of melatonin as a signal molecule, likely related to fermentation metabolism [17,24]. Therefore, the quantity of sugar present in the medium, which can determine the metabolism of sugars in yeast, could be a key parameter in melatonin synthesis or function.

### 3.3. Effect of Temperature on Melatonin Synthesis

To elucidate the effect of temperature in melatonin production, alcoholic fermentations were performed at 4 and 12 °C, two extreme temperatures, in which a longer lag phase and therefore, a delay in the alcoholic fermentation is expected, in comparison to standard fermentation carried out at 28 °C.

Intracellular melatonin was detected at all temperatures tested (Figure 4, Table S3). However, surprisingly, higher differences were observed in melatonin synthesis at 12 than 4 °C (Figure 5, Tables S9 and S10). On melatonin production profile, it peaked at 240 min at 12 °C, whereas at the other two temperatures, the highest synthesis was observed at 60 min. Additionally, another peak of lower concentration appeared at 120 min at both low temperatures (4 and 12 °C). Temperature is an important factor during alcoholic fermentation that affects its development, as well as yeast growth, being slower at lower temperatures [9,10]. The results at 12 °C seemed to point towards a delayed effect on melatonin synthesis due to the prolonged lag phase at low temperatures. In fact, Wang et al. [28] observed that fermentations carried out at 16 °C delayed and decreased melatonin production in mulberry wines. However, the results at 4 °C did not support this delay effect; it may be that extreme temperatures such as 4 °C triggered other mechanisms responsible for this early melatonin synthesis.

### 3.4. Effect of the Initial Yeast Population on Melatonin Synthesis

To understand the effect of inoculum amount in melatonin production, fermentations with 10^7^ and 10^8^ cells/mL were carried out in comparison with the standard inoculation rate (10^6^ cell/mL). As mentioned above, in standard inoculation conditions, melatonin was detected at 60 min (Figure 4). When the inoculum was increased, melatonin synthesis was delayed at 140 min for 10^7^ cell/mL and 180 min for 10^8^ cell/mL. Therefore, the higher the population we used, the greater the delay of the intracellular melatonin peak we obtained. Additionally, melatonin content was higher in 10^7^ cell/mL inoculum (Figure 5, Tables S3, S9 and S10). Given that high inoculum size causes less growth of cells [35], this delay in melatonin synthesis may add more evidences that point out melatonin as a signal molecule for yeast growth.

### 3.5. Effect of Cell Cycle Synchronization on Melatonin Synthesis

Finally, as we obtained high variability in melatonin concentration between triplicates, we wanted to synchronize the cell cycle to have most cells in the same cell cycle phase and thus decrease this variability. Due to the impossibility of synchronizing QA23, the correspondent haploid was studied (QA23 ho-). Treatment with alpha-factor was used to synchronize the cell cycle in G1 phase.

However, despite synchronizing 85% of cells, we did not achieve a clear decrease in melatonin detection variability (Figure 5, Tables S4, S9 and S10), probably due to its fast appearance and disappearance and the impossibility of monitoring its synthesis in smaller periods.

## 4. Conclusions

Although differences were observed between strains, in this study, we can confirm that the pattern of melatonin synthesis described for the strain *S. cerevisiae* QA23 in a previous study [17] is also observed in the rest of strains of *Saccharomyces.* Therefore, the production of intracellular melatonin during the lag phase of yeast growth and its secretion to the medium in the mid-end exponential or early stationary phases seem to be a species-specific pattern and not strain dependent. Moreover, differences between strains can be attributed to their different isolation origin or uses, and therefore, to different adaptation mechanisms to the fermentation medium, as Vigentini et al. [14] described for *Torulaspora delbrueckii* and *S. cerevisiae* strains.

When several conditions that affect the performance of alcoholic fermentation were tested, the intracellular melatonin peak was delayed in a low sugar concentration, a fermentation temperature of 12 °C and with a higher initial population. Nevertheless, in all conditions, melatonin appeared during lag phase and as a rapid signal molecule. It is also important to highlight that melatonin quantities were very different between conditions, occurring the highest production when the fermentation temperature was fixed at 12 °C.

Finally, despite the fact that we were not able to clearly unravel which conditions trigger melatonin synthesis in yeast cells during alcoholic fermentation, we observed that sugar content has the most apparent effect on melatonin synthesis. These results together with previous ones of the group, in which interactions of melatonin with glycolytic proteins were described [17,24], seem to clearly indicate an active role of this molecule in the sugar metabolism, specifically in fermentative metabolism. Nevertheless, further studies are needed to confirm this hypothesis and to understand the exact mechanism and the triggering of melatonin synthesis.

## Supplementary Materials

The following are available online at https://www.mdpi.com/2076-2607/8/6/853/s1, Table S1: Intracellular and extracellular melatonin produced by the four different *Saccharomyces* strains during alcoholic fermentation, Table S2: Intracellular melatonin production by *S. cerevisiae* QA23 in a standard, low-glucose and -nitrogen grape must, Table S3: Effect of low temperature and inoculum size on intracellular melatonin production by *S. cerevisiae* QA23, Table S4: Comparison of intracellular melatonin production by arrested and non-arrested yeast cells, Table S5: Analysis of differences between intracellular melatonin synthesis by different *Saccharomyces* strains, Table S6: Analysis of differences between intracellular melatonin synthesis profile by different *Saccharomyces* strains, Table S7: Analysis of differences between extracellular melatonin synthesis by different *Saccharomyces* strains, Table S8: Analysis of differences between extracellular melatonin synthesis profile by different *Saccharomyces* strains, Table S9: Analysis of differences between intracellular melatonin synthesis on different conditions, Table S10: Analysis of differences between intracellular melatonin synthesis profile on different conditions.
