# Supplementary material for: Effect of Several Nutrients and Environmental Conditions on Intracellular Melatonin Synthesis in Saccharomyces cerevisiae
Source: Microorganisms. 2020 Jun 5;8(6):853. doi: 10.3390/microorganisms8060853 (PMC7355912; doi:10.3390/microorganisms8060853)
Supplement: Supplementary file 1 [file microorganisms-08-00853-s001.pdf]

Supplementary material

Supplementary Table 1: Intracellular and extracellular melatonin produced by the four different *Saccharomyces* strains during alcoholic fermentation. Data are expressed in ng/mL.

n.d.: not detected; I: Intracellular; E: Extracellular.

Yeast strain		Time (h)											
		1	4	8	12	15	24	30	36	48	72	96	168
QA23	I	1.38±0.15	0.90±0.16	0.40±0.04	0.20±0.07	0.26±0.00	0.28±0.04	n.d.	0.01±0.01	n.d.	n.d.	0.02±0.02	0.01±0.01
	E	0.16±0.00	0.13±0.02	0.16±0.03	0.31±0.02	0.27±0.22	1.35±1.69	0.28±0.24	0.36±0.28	0.15±0.00	1.09±1.34	0.25±0.07	0.18±0.08
Instaferm	I	0.48±0.01	1.47±1.17	0.28±0.01	0.25±0.08	0.07±0.09	0.21±0.04	n.d.	0.01±0.01	0.01±0.01	0.05±0.06	0.01±0.01	n.d.
	E	0.19±0.00	0.52±0.11	0.38±0.31	0.56±0.46	0.46±0.36	0.82±0.32	0.84±0.72	0.97±0.47	1.07±1.02	1.37±1.61	1.48±1.88	1.82±2.37
Levucell	I	51.80±0.01	67.44±4.91	27.57±3.02	25.97±1.54	20.16±2.35	22.70±2.08	17.53±1.61	17.03±0.05	19.17±1.48	16.67±0.23	17.63±1.78	18.89±0.25
	E	0.10±0.00	0.17±0.03	0.17±0.04	0.19±0.02	0.33±0.29	93.14±85.86	0.87±0.48	0.39±0.15	2.34±2.57	0.44±0.38	0.33±0.34	0.60±0.53
Diamond	I	46.97±0.50	52.56±4.31	26.08±3.37	20.54±3.15	19.57±5.83	21.38±3.40	15.77±2.24	17.95±1.09	18.33±0.58	17.26±023	12.82±2.92	12.73±0.28
	E	0.07±0.00	0.10±0.06	0.21±0.11	0.07±0.00	0.05±0.01	11.14±1.07	6.47±8.76	0.95±1.05	16.13±12.73	0.10±0.01	1.00±1.25	0.15±0.03

Supplementary Table 2: Intracellular melatonin production by *S. cerevisiae* QA23 in a standard, low-glucose and low-nitrogen grape must. Data are expressed in ng/mL per 10^8 cells.

Time (min)	Standard*	Low glucose	Low nitrogen
0	n.d.	n.d.	n.d.
30	n.d.	0.55±0.45	0.13±0.22
40	n.d.	0.01±0.01	3.85±3.38
50	n.d.	n.d.	8.79±9.26
60	2.34±0.37	0.95±1.00	0.59±0.61
70	0.01±0.02	n.d.	8.51±10.32
80	0.01±0.01	0.01±0.01	9.59±16.61
90	n.d.	3.19±4.49	0.07±0.13
100	n.d.	n.d.	n.d.
110	n.d.	4.11±1.00	n.d.
120	n.d.	4.20±3.36	0.02±0.02
130	n.d.	1.41±2.00	n.d.
140	n.d.	1.13±1.59	n.d.
150	0.01±0.01	7.77±5.15	0.11±0.19
160	0.56±0.19	3.80±0.15	n.d.
170	n.d.	3.80±0.05	n.d.
180	0.45±0.12	3.34±3.38	0.08±0.14
190	0.01±0.01	7.45±9.26	n.d.
200	n.d.	5.07±0.86	n.d.
210	n.d.	8.18±6.85	n.d.
220	n.d.	3.56±0.12	0.32±0.56
230	n.d.	3.58±0.05	n.d.
240	n.d.	0.82±1.42	0.17±0.29

n.d. not detected

*Standard was Sugar 200 g/L and nitrogen 300 mg/L, inoculated at 10^6 cells/mL and fermented at 28°C.

Supplementary Table 3. Effect of low temperature and inoculum size on intracellular melatonin production by *S. cerevisiae* QA23. Data are expressed in ng/mL per 10^8 cells.

Time (min)	Standard*	4ºC	12ºC	10^7 cells/mL	10^8 cells/mL
0	n.d.	n.d.	n.d.	n.d.	n.d.
30	n.d.	n.d.	n.d.	n.d.	n.d.
40	n.d.	n.d.	n.d.	n.d.	0.13±0.02
50	n.d.	n.d.	n.d.	n.d.	n.d.
60	2.34±0.37	6.01±9.82	0.40±0.70	n.d.	0.24±0.03
70	0.01±0.02	0.01±0.01	n.d.	n.d.	n.d.
80	0.01±0.01	0.01±0.01	n.d.	n.d.	n.d.
90	n.d.	0.05±0.01	0.23±0.40	n.d.	n.d.
100	n.d.	n.d.	0.50±0.45	n.d.	n.d.
110	n.d.	n.d.	n.d.	n.d.	n.d.
120	n.d.	5.35±9.27	462.40±800.45	n.d.	n.d.
130	n.d.	n.d.	0.01±0.02	n.d.	n.d.
140	n.d.	n.d.	n.d.	24.33±2.92	n.d.
150	0.01±0.01	n.d.	2.78±0.70	n.d.	n.d.
160	0.56±0.19	n.d.	0.23±0.11	n.d.	n.d.
170	n.d.	n.d.	0.11±0.06	n.d.	n.d.
180	0.45±0.12	n.d.	27.70±1.02	n.d.	0.87±0.15
190	0.01±0.01	n.d.	0.01±0.01	n.d.	n.d.
200	n.d.	n.d.	n.d.	n.d.	n.d.
210	n.d.	n.d.	0.63±1.06	n.d.	n.d.
220	n.d.	n.d.	n.d.	2.06±0.52	n.d.
230	n.d.	n.d.	0.26±0.25	n.d.	n.d.
240	n.d.	n.d.	1328.92±0.69	n.d.	n.d.

n.d. not detected

*Standard was Sugar 200 g/L and nitrogen 300 mg/L, inoculated at 10^6 cells/mL and fermented at 28°C.

Supplementary Table 4: Comparison of intracellular melatonin production by arrested and non-arrested yeast cells. Data are expressed in ng/mL per 10^8 cells.

Time (min)	Non-arrested	Arrested
0	n.d.	n.d.
30	n.d.	n.d.
40	n.d.	n.d.
60	7.88±1.67	16.87±23.86
80	12.58±4.83	5.79±0.62
100	4.40±1.14	20.53±22.55
120	21.82±12.42	8.49±2.01
140	7.43±0.89	6.95±0.40
160	0.01±0.01	n.d.
180	4.96±0.37	8.79±11.19
200	n.d.	n.d.
220	n.d.	n.d.
240	n.d.	n.d.

n.d. not detected

Supplementary Table 5: Tukey (HSD)/ Analysis of differences between intracellular melatonin synthesis by different *Saccharomyces* strains. (Confidence 95%).

**Contrast**	**Difference**	**Standard difference**	**Critical value**	**p.value**	**Significance**
QA23 vs Diamond	-2,262	-4,841	2,675	**0,000**	Yes
QA23 vs Levucell	-1,546	-3,309	2,675	**0,010**	Yes
QA23 vs Instaferm	-0,061	-0,130	2,675	0,999	No
Instaferm vs Diamond	-2,201	-4,819	2,675	**0,000**	Yes
Instaferm vs Levucell	-1,485	-3,252	2,675	**0,012**	Yes
Levucell vs Diamond	-0,716	-1,567	2,675	0,408	No
Tukey critical d value:			3,783		

**Category**	**Medium LS**	**Standard error**	**Groups**	
QA23	0,056	0,337	A	
Instaferm	0,117	0,323	A	
Levucell	1,602	0,323		B
Diamond	2,318	0,323		B

Supplementary Table 6: Tukey (HSD)/ Analysis of differences between intracellular melatonin synthesis profile by different *Saccharomyces* strains. (Confidence 95%).

Contrast	Difference	Standard difference	Critical value	p.value	Significance
Diamond vs Instaferm	28,409	1,485	2,675	0,456	No
Diamond vs QA23	27,023	1,367	2,675	0,526	No
Diamond vs Levucell	4,809	0,274	2,675	0,993	No
Levucell vs Instaferm	23,600	1,265	2,675	0,590	No
Levucell vs QA23	22,214	1,154	2,675	0,659	No
QA23 vs Instaferm	1,386	0,077	2,675	1,000	No
Tukey critical d value:			3,783		

Category	Medium LS	Standard error	Groups
Diamond	58,529	13,099	A
Levucell	53,720	12,738	A
QA23	31,506	13,575	A
Instaferm	30,120	12,861	A

Supplementary Table 7: Tukey (HSD)/ Analysis of differences between extracellular melatonin synthesis by different *Saccharomyces* strains. (Confidence 95%).

Contrast	Difference	Standard difference	Critical value	p.value	Significance
QA23 vs Levucell	-7,152	-1,338	2,675	0,544	No
QA23 vs Diamond	-1,685	-0,315	2,675	0,989	No
QA23 vs Instaferm	-0,397	-0,074	2,675	1,000	No
Instaferm vs Levucell	-6,755	-1,293	2,675	0,573	No
Instaferm vs Diamond	-1,288	-0,246	2,675	0,995	No
Diamond vs Levucell	-5,468	-1,046	2,675	0,723	No
Tukey critical d value:			3,783		

Category	Medium LS	Standard error	Groups
QA23	0,395	3,861	A
Instaferm	0,792	3,695	A
Diamond	2,080	3,695	A
Levucell	7,547	3,695	A

Supplementary Table 8: Tukey (HSD)/ Analysis of differences between extracellular melatonin synthesis profile by different *Saccharomyces* strains. (Confidence 95%).

**Contrast**	**Difference**	**Standard difference**	**Critical value**	**p.value**	**Significance**
Levucell vs Instaferm	-39,025	-3,196	2,675	**0,014**	Sí
Levucell vs QA23	-21,161	-1,694	2,675	0,339	No
Levucell vs Diamond	-9,957	-0,816	2,675	0,847	No
Diamond vs Instaferm	-29,067	-2,381	2,675	0,096	No
Diamond vs QA23	-11,204	-0,897	2,675	0,806	No
QA23 vs Instaferm	-17,863	-1,430	2,675	0,488	No
Tukey critical d value:			3,783		

**Category**	**Medium LS**	**Standard error**	**Groups**	
Levucell	8,952	8,633	A	
Diamond	18,909	8,633	A	B
QA23	30,113	9,021	A	B
Instaferm	47,977	8,633		B

Supplementary Table 9: Fisher (LSD)/ Analysis of differences between intracellular melatonin synthesis by *S. cerevisiae* QA23 on different conditions: nutrients, temperature, inoculum and synchronization. (Confidence 95%).

**Contrast**	**Difference**	**Standard difference**	**Critical value**	**p.value**	**Significance**
Temperature 12ºC vs 108 cells/mL	1327,623	30,332	2,101	**<0,0001**	Yes
Temperature 12ºC vs Temperature 4ºC	1322,472	30,214	2,101	**<0,0001**	Yes
Temperature 12ºC vs Sugar 20 g/L	1320,303	30,164	2,101	**<0,0001**	Yes
Temperature 12ºC vs Nitrogen 100 mg/L	1318,897	30,132	2,101	**<0,0001**	Yes
Temperature 12ºC vs Arrested cells	1314,803	30,039	2,101	**<0,0001**	Yes
Temperature 12ºC vs Non-arrested cells	1313,940	30,019	2,101	**<0,0001**	Yes
Temperature 12ºC vs 107 cells/mL	1304,153	29,795	2,101	**<0,0001**	Yes
Temperature 12ºC vs Sugar 200 g/L	1234,022	28,193	2,101	**<0,0001**	Yes
Sugar 200 g/L vs 108 cells/mL	93,602	2,138	2,101	**0,046**	Yes
Sugar 200 g/L vs Temperature 4ºC	88,451	2,021	2,101	0,058	No
Sugar 200 g/L vs Sugar 20 g/L	86,282	1,971	2,101	0,064	No
Sugar 200 g/L vs Nitrogen 100 mg/L	84,875	1,939	2,101	0,068	No
Sugar 200 g/L vs Arrested cells	80,781	1,846	2,101	0,081	No
Sugar 200 g/L vs Non-arrested cells	79,918	1,826	2,101	0,085	No
Sugar 200 g/L vs 107 cells/mL	70,132	1,602	2,101	0,126	No
107 cells/mL vs 108 cells/mL	23,470	0,536	2,101	0,598	No
107 cells/mL vs Temperature 4ºC	18,319	0,419	2,101	0,681	No
107 cells/mL vs Sugar 20 g/L	16,150	0,369	2,101	0,716	No
107 cells/mL vs Nitrogen 100 mg/L	14,744	0,337	2,101	0,740	No
107 cells/mL vs Arrested cells	10,650	0,243	2,101	0,811	No
107 cells/mL vs Non-arrested cells	9,786	0,224	2,101	0,826	No
Non-arrested cells vs 108 cells/mL	13,684	0,313	2,101	0,758	No
Non-arrested cells vs Temperature 4ºC	8,533	0,195	2,101	0,848	No
Non-arrested cells vs Sugar 20 g/L	6,364	0,145	2,101	0,886	No
Non-arrested cells vs Nitrogen 100 mg/L	4,957	0,113	2,101	0,911	No
Non-arrested cells vs Arrested cells	0,863	0,020	2,101	0,984	No
Arrested cells vs 108 cells/mL	12,820	0,293	2,101	0,773	No
Arrested cells vs Temperature 4ºC	7,669	0,175	2,101	0,863	No
Arrested cells vs Sugar 20 g/L	5,500	0,126	2,101	0,901	No
Arrested cells vs Nitrogen 100 mg/L	4,094	0,094	2,101	0,927	No
Nitrogen 100 mg/L vs 108 cells/mL	8,726	0,199	2,101	0,844	No
Nitrogen 100 mg/L vs Temperature 4ºC	3,575	0,082	2,101	0,936	No
Nitrogen 100 mg/L vs Sugar 20 g/L	1,406	0,032	2,101	0,975	No
Sugar 20 g/L vs 108 cells/mL	7,320	0,167	2,101	0,869	No
Sugar 20 g/L vs Temperature 4ºC	2,169	0,050	2,101	0,961	No
Temperature 4ºC vs 108 cells/mL	5,151	0,118	2,101	0,908	No
LSD-valor:			91,958		

Condition	Medias LS (Melatonin)	Standard error	Groups		
Temperature 12ºC	1328,487	30,950	A		
Sugar 200 g/L	94,465	30,950		B	
107 cells/mL	24,333	30,950		B	C
Non-arrested cells	14,547	30,950		B	C
Arrested cells	13,684	30,950		B	C
Nitrogen 100 mg/L	9,590	30,950		B	C
Sugar 20 g/L	8,183	30,950		B	C
Temperature 4ºC	6,014	30,950		B	C
108 cells/mL	0,863	30,950			C

Supplementary Table 10: Tukey (HSD)/ Analysis of differences between intracellular melatonin synthesis profile by *S. cerevisiae* QA23 on different conditions: nutrients, temperature, inoculum and synchronization. (Confidence 95%).

Contrast	Difference	Standard difference	Critical value	p.value	Significance
107 cells/mL vs Sugar 20 g/L	-27,363	-4,085	3,250	**0,003**	Yes
107 cells/mL vs Arrested cells (ho-)	-8,798	-1,328	3,250	0,963	No
107 cells/mL vs Nitrogen 100 mg/L	-8,774	-1,325	3,250	0,964	No
107 cells/mL vs Not arrested (ho-)	-6,489	-0,980	3,250	0,996	No
107 cells/mL vs Temperature 28ºC	-5,635	-0,851	3,250	0,999	No
107 cells/mL vs Temperature 4ºC	-3,222	-0,486	3,250	1,000	No
107 cells/mL vs Sugar 200 g/L	-2,524	-0,381	3,250	1,000	No
107 cells/mL vs Temperature 12ºC	-2,181	-0,329	3,250	1,000	No
107 cells/mL vs 108 cells/mL	-1,376	-0,208	3,250	1,000	No
108 cells/mL vs Sugar 20 g/L	-25,987	-3,879	3,250	**0,006**	Yes
108 cells/mL vs Arrested cells (ho-)	-7,422	-1,121	3,250	0,989	No
108 cells/mL vs Nitrogen 100 mg/L	-7,398	-1,117	3,250	0,990	No
108 cells/mL vs Not arrested (ho-)	-5,113	-0,772	3,250	1,000	No
108 cells/mL vs Temperature 28ºC	-4,259	-0,643	3,250	1,000	No
108 cells/mL vs Temperature 4ºC	-1,846	-0,279	3,250	1,000	No
108 cells/mL vs Sugar 200 g/L	-1,148	-0,173	3,250	1,000	No
108 cells/mL vs Temperature 12ºC	-0,804	-0,121	3,250	1,000	No
Temperature 12ºC vs Sugar 20 g/L	-25,182	-3,759	3,250	**0,010**	Yes
Temperature 12ºC vs Arrested cells (ho-)	-6,617	-0,999	3,250	0,996	No
Temperature 12ºC vs Nitrogen 100 mg/L	-6,593	-0,995	3,250	0,996	No
Temperature 12ºC vs Not arrested (ho-)	-4,308	-0,651	3,250	1,000	No
Temperature 12ºC vs Temperature 28ºC	-3,454	-0,522	3,250	1,000	No
Temperature 12ºC vs Temperature 4ºC	-1,041	-0,157	3,250	1,000	No
Temperature 12ºC vs Sugar 200 g/L	-0,343	-0,052	3,250	1,000	No
Sugar 200 g/L vs Sugar 20 g/L	-24,839	-3,708	3,250	**0,011**	Yes
Sugar 200 g/L vs Arrested cells (ho-)	-6,274	-0,947	3,250	0,997	No
Sugar 200 g/L vs Nitrogen 100 mg/L	-6,250	-0,944	3,250	0,997	No

Contrast	Difference	Standard difference	Critical value	p.value	Significance
Sugar 200 g/L vs Not arrested (ho-)	-3,965	-0,599	3,250	1,000	No
Sugar 200 g/L vs Temperature 28ºC	-3,111	-0,470	3,250	1,000	No
Sugar 200 g/L vs Temperature 4ºC	-0,698	-0,105	3,250	1,000	No
Temperature 4ºC vs Sugar 20 g/L	-24,141	-3,604	3,250	**0,016**	Yes
Temperature 4ºC vs Arrested cells (ho-)	-5,576	-0,842	3,250	0,999	No
Temperature 4ºC vs Nitrogen 100 mg/L	-5,552	-0,838	3,250	0,999	No
Temperature 4ºC vs Not arrested (ho-)	-3,267	-0,493	3,250	1,000	No
Temperature 4ºC vs Temperature 28ºC	-2,413	-0,364	3,250	1,000	No
Temperature 28ºC vs Sugar 20 g/L	-21,728	-3,244	3,250	0,051	No
Temperature 28ºC vs Arrested cells (ho-)	-3,163	-0,478	3,250	1,000	No
Temperature 28ºC vs Nitrogen 100 mg/L	-3,139	-0,474	3,250	1,000	No
Temperature 28ºC vs Not arrested (ho-)	-0,854	-0,129	3,250	1,000	No
Not arrested (ho-) vs Sugar 20 g/L	-20,874	-3,116	3,250	0,073	No
Not arrested (ho-) vs Arrested cells (ho-)	-2,309	-0,349	3,250	1,000	No
Not arrested (ho-) vs Nitrogen 100 mg/L	-2,285	-0,345	3,250	1,000	No
Nitrogen 100 mg/L vs Sugar 20 g/L	-18,589	-2,775	3,250	0,174	No
Nitrogen 100 mg/L vs Arrested cells (ho-)	-0,024	-0,004	3,250	1,000	No
Arrested cells (ho-) vs Sugar 20 g/L	-18,565	-2,771	3,250	0,176	No
Tukey critical d value:			4,596		

Category	Medium LS	Standard error	Groups	
107 cells/mL	4,339	4,683	A	
108 cells/mL	5,715	4,683	A	
Temperature 12ºC	6,520	4,683	A	
Sugar 200 g/L	6,863	4,683	A	
Temperature 4ºC	7,561	4,683	A	
Not arrested (ho-)	10,828	4,683	A	B
Nitrogen 100 mg/L	13,113	4,683	A	B
Arrested cells (ho-)	13,137	4,683	A	B
Sugar 20 g/L	31,702	4,789		B
